# How to Relax in Stressful Situations: A Smart Stress Reduction System

**DOI:** 10.3390/healthcare8020100

**Published:** 2020-04-16

**Authors:** Yekta Said Can, Heather Iles-Smith, Niaz Chalabianloo, Deniz Ekiz, Javier Fernández-Álvarez, Claudia Repetto, Giuseppe Riva, Cem Ersoy

**Affiliations:** 1 Computer Engineering Department, Bogazici University, 34342 Istanbul, Turkey; niaz.chalabianloo@boun.edu.tr (N.C.); deniz.ekiz@boun.edu.tr (D.E.); ersoy@boun.edu.tr (C.E.); 2 Leeds Teaching Hospitals NHS Trust/University of Leeds, Leeds LS1 3EX, UK; heather.iles-smith@nhs.net; 3 General Psychology and Communication Psychology, Catholic University of Milan, 20123 Milan, Italy; javier.fernandezkirszman@unicatt.it (J.F.-Á.); claudia.repetto@unicatt.it (C.R.); giuseppe.riva@unicatt.it (G.R.)

**Keywords:** commercial smartwatch, mental stress, psychophysiological, emotion regulation, heart rate variability, electrodermal activity

## Abstract

Stress is an inescapable element of the modern age. Instances of untreated stress may lead to a reduction in the individual’s health, well-being and socio-economic situation. Stress management application development for wearable smart devices is a growing market. The use of wearable smart devices and biofeedback for individualized real-life stress reduction interventions has received less attention. By using our unobtrusive automatic stress detection system for use with consumer-grade smart bands, we first detected stress levels. When a high stress level is detected, our system suggests the most appropriate relaxation method by analyzing the physical activity-based contextual information. In more restricted contexts, physical activity is lower and mobile relaxation methods might be more appropriate, whereas in free contexts traditional methods might be useful. We further compared traditional and mobile relaxation methods by using our stress level detection system during an eight day EU project training event involving 15 early stage researchers (mean age 28; gender 9 Male, 6 Female). Participants’ daily stress levels were monitored and a range of traditional and mobile stress management techniques was applied. On day eight, participants were exposed to a ‘stressful’ event by being required to give an oral presentation. Insights about the success of both traditional and mobile relaxation methods by using the physiological signals and collected self-reports were provided.

## 1. Introduction

Stress constitutes a complex process that is activated by a physical or mental threat to the individuals’ homeostasis, comprising a set of diverse psychological, physiological and behavioral responses [1]. Although it is usually considered a negative response, stress actually constitutes a key process for ensuring our survival. However, when a stress response is repeatedly triggered in the absence of a challenging stimulus, or if there is constant exposure to challenging situations, stress can become harmful. Evidence suggests that, in either of these two contexts, stress is a persistent factor for the development of psycho-pathological conditions [2,3].

When faced with stressful events, people make autonomic and controlled efforts to reduce the negative impact and maximize the positive impact that every specific situation may provoke. Generally, this process is denominated as emotion regulation, formally defined as the process by which individuals can influence what emotions they have, when they have them and how they experience and express those emotions [4]. It has been suggested that the term emotion regulation can be understood as a broad tag that comprises the regulation of all responses that are emotionally charged, from basic emotions to complex mood states as well as regulation of everyday life [5].

Failure to address triggers of stress has been shown to lead to chronic stress, anxiety and depression, and attributed to serious physical health conditions such as cardiovascular disease [6]. The World Health Organization concluded that psychological stress is one of the most significant health problems in the 21st-century and is a growing problem [7]. There are various interventions to minimize stress based on individual preferences and requirements. Stress management techniques including ancient practices such as Tai Chi [8] and yoga [9] as well as other physical activities [10] are often cited as being helpful in combating stress. Likewise traditional meditation, mindfulness [11] and cognitive behavioural therapy (CBT) [12] all have established benefits. These techniques are not applicable in office or social environments, or during most daily routines. Therefore, a smart device based stress management application may be of benefit. Recently, smartphone applications such as Calm, Pause, Heartmath and Sway have been developed for indoor environments. However, these applications are not individualized nor do they include biofeedback and studies that validate their effects are limited [13].

In this study, we used the stress level detection scheme using physiological signals and added a physical activity based context analyzer. When the user experiences a high stress level, the system suggests appropriate stress reduction methods (traditional or mobile). We further compare the effects of traditional and mobile stress alleviation methods on physiological data of 15 international Ph.D. students (participants) during eight days of training. In addition, 1440 h of physiological signals from Empatica E4 smart bands were collected in this training event. Stress management techniques based on the emotion regulation model of James Gross [4] were applied to reduce participant stress levels. To the best of our knowledge, this work is the first one suggesting appropriate stress reduction methods based on contextual information and comparing both traditional and mobile stress management interventions in the real-life environment using a commercial smart-band based automatic stress level detection system that eliminates motion artifacts. Using such a system is essential because these offline stress level detection algorithms could be used in real-time biofeedback apps.

Application of our stress level detection algorithm, in a real world context, could allow individuals to receive feedback regarding high stress levels along with recommendations for relaxation methods. Additional continued monitoring may also enable the individual to better understand the effectiveness of any stress reduction methods. However, for our stress detection algorithm to be applied in daily life, the smart device should be unobtrusive (i.e., should not be comprised of cables, electrodes, boards). Our system works on smart-bands which are perfect examples of this type of unobtrusive wearable device.

This paper describes emotion regulation in the context of stress management and how yoga and mindfulness can be used for regulating emotions (Section 2). Methods of detecting stress and analyzing context based on physical activity are described (Section 3) and data are presented related to our method for stress level detection with the use of smart-bands (Section 4). Experimental results and discussion are also presented (Section 5) and we present the conclusions and future works of the study (Section 6).

The major research contributions of this study are the following:

- Developing a physical activity based context analyzer and relaxation method suggestion system
- Comparison of stress reduction methods (mobile mindfulness, traditional mindfulness and yoga) and their effectiveness in the context of stress management with the use of an unobtrusive smartwatch based stress level detection system
- Application of James Gross’s prominent emotion regulation model in the context of stress management and measuring the physiological component with smart bands.

## 2. Background

### 2.1. Emotion Regulation in the Context of Stress Management

Stress is a normal part of daily life. However, its effects often vary across individuals and despite similar circumstances, some people do not feel under strain while others may be severely affected. Multiple reasons exist for these differences between individuals, including how people perceive reality and how they respond to the numerous stimuli to which they are exposed. When a person believes that a certain situation surpasses their available coping mechanisms, it is referred to as perceived stress. Thus, perceived stress varies from person to person depending on the value that an individual gives to a situation and their self-recognition of the resources to deal with it.

Numerous psychological scientists have investigated perceived stress. Individuals who display a mismatch between contextual demands and perceived resources constantly (rather than during a specific moment in time) are referred to as experiencing chronic stress. Chronic stress has not only been shown to be very relevant in people’s well-being and quality of life, but also important in the appearance and maintenance of several physical and mental diseases [14].

As a consequence, mounting research has focused on the mechanisms that people implement in order to alleviate the physical and cognitive burden associated with that perceived stress. Coping styles, stress management techniques, self-regulation, or emotion regulation techniques are different labels that define the way people implement certain behavioral, cognitive, or emotional strategies to maintain allosteric load [15]. In other words, every living organism needs to vary among plasticity and stability in order to survive. Human beings are not the exception to the rule and the complex system that applies to every single person and the necessity of reaching a constant level of regulation permits the individuals to pursue their goals.

Specifically, emotion regulation has been defined as the study of “the processes by which we influence which emotions we have when we have them, and how we experience and express them” [4]. A large body of evidence has shown that there are very different consequences depending on the effectiveness people achieve to regulate their emotions. Naturally, both at an implicit or explicit level, people regulate emotions in order to maintain those allosteric levels previously mentioned. Therefore, when there are specific stressors that demand a particular cognitive or physical response, the emotional reactivity may be stronger and the need for a proper regulation more relevant. Indeed, emotion regulation has shown to be a transdiagnostic factor that is present at a wide range of mental disorders. In other words, the way people initiate, implement and monitor their emotional processes, in order to reach more desirable states, has a significant impact on the stress levels. Some emotion regulation (ER) strategies have shown to be correlated with mental health issues. Among these strategies, cognitive reappraisal, problem-solving, or acceptance shall be mentioned as strategies that are negatively correlated with psychopathology, while rumination, experiential avoidance, or suppression are positively correlated with psychopathology [16]. In this regard, hinging on the different ER strategies deployed, ER can constitute a protective factor to face stress responses that all individuals experience after minor or major stressors [17]. Additionally, an adaptive regulation of emotions, by managing stress, may also be beneficial for clinical populations, such as people suffering from affective disorders [18,19].

Therefore, from whole psychotherapeutic treatments to single self-applied applications, studies in the literature have focused on how people can better regulate their emotions and manage their stress levels. Among many other techniques, cognitive behavioral therapy, autogenic training, biofeedback, breathing exercises, relaxation techniques, guided imagery, mindfulness, yoga, or Tai-Chi, are some of the stress management interventions that have received attention from researchers [20,21].

### 2.2. Yoga and Mindfulness: As Tools for Emotion Regulation

#### 2.2.1. Yoga

Yoga is an ancient Eastern practice that developed more than 2000 years ago. Although its original creator and source are uncertain, the earliest written word ‘Yoga Sutra’ describes the philosophy of yoga focussing on growing spirituality, regulating emotions and thoughts. Initially, the focus was on awareness of breathing and breathing exercises ‘pranayama’ to calm the mind and body, ultimately reaching a higher state of consciousness.

As yoga evolved, physical movement in the form of postures was included and integrated with yogic breathing ‘prana’ and elements of relaxation. The underlying purpose is to create physical flexibility, reduce pain and unpleasant stimuli and reduce negative thoughts and emotions to calm the mind and body, thereby improving well-being. In the healthcare literature, the benefits are reported to be far-reaching both for mental and physical health conditions such as anxiety, depression, cardiovascular disease, cancer and respiratory symptoms. It is also reported to reduce muscular-skeletal problems and physical symptoms through increasing the awareness of the physical body.

Yoga has become a global phenomenon and is widely practiced in many different forms. Generally, all types of yoga include some elements of relaxation. Additionally, some forms include mainly pranayama and others are more physical in nature. One such practice is vinyasa flow which involves using the inhale and exhale of the breathing pattern to move through a variety of yoga postures; this leads to the movement becoming meditative. The practice often includes pranayama followed by standing postures linked together with a movement called vinyasa, (similar to a sun salutation) which helps to keep the body moving and increases fitness, flexibility and helps maintain linkage with the breath. The practice also often includes a range of seated postures, an inversion (such as headstand or shoulder stand) and final relaxation ‘savasana’.

#### 2.2.2. Mindfulness

Mindfulness involves being more present at the moment by acknowledging the here and now, often referred to as ‘being present’ rather than focussing on the past or future [8]. Being present may include being aware of our surroundings and the environment, or of what we are eating and drinking and physical sensations such as the sun or wind on our skin.

Acknowledging the thoughts and body are also aspects of mindfulness. Each day humans experience thousands of thoughts, the majority being of no consequence. In some instances, these thoughts are repetitive and negative in nature which can lead to increased stress and the related unpleasant physical symptoms such as feeling anxious, nausea and tension headaches. Being mindful includes an awareness of our thinking and whether we are caught up with our thoughts rather than being aware of the moment. Additionally, on a daily basis, awareness of the physical body may be minimal; being mindful includes increasing this awareness through becoming more connected with the sensations in the body. This might include experiencing the legs moving when walking, or feeling the ground under the feet or the natural way of the body whilst standing.

Mindfulness has been shown to be of benefit to physical and mental health. It is currently recommended by the National Institute for Clinical Excellence [22] as adjunctive therapy to Cognitive Behavioural Therapy (CBT) for the prevention of relapse depression.

However, it may be challenging for some individuals to do this with a multitude of distractions around them and, therefore, they may choose to identify a particular time and place when and where they can sit in a comfortable position to start to become aware of their breathing and bodily sensations.

#### 2.2.3. Mobile Mindfulness Inspired By Tai-Chi—Pause

Tai-Chi is an internal Chinese martial art practiced for both its defense training, its health benefits and meditation. There is good evidence of benefits for depression, cardiac and stroke rehabilitation and dementia [23]. The term Tai-Chi refers to a philosophy of the forces of yin and yang, related to the moves. An iPhone application Pause inspired by Tai-Chi is used for guided mindfulness which draws upon the principles of mindfulness meditation to trigger the body’s rest and digest response, quickly restoring attention [24].

## 3. Related Work

Researchers have created the ability to detect stress in laboratory environments with medical-grade devices [25,26,27,28]; smartwatches and smart bands started to be used for stress level detection studies [29,30,31]. These devices provide high comfort and rich functionality for the users, but their stress detection accuracies are lower than medical-grade devices due to low signal quality and difficulty obtaining data in intense physical activity. If data are collected for long periods, researchers have shown that their detection performance improves [32]. During movement periods, the signal can be lost (gap in the data) or artifacts might be generated. Stress level detection accuracies for 2-classes by using these devices are around 70% [29,30,33,34].

After detecting the stress level of individuals, researchers should recover from the stressed state to the baseline state. To the best of our knowledge, there are very few studies that combine automatic stress detection (using physiological data) with recommended appropriate stress management techniques. Ahani et al. [35] examined the physiological effect of mindfulness. They used the Biosemi device which acquires electroencephalogram (EEG) and respiration signals. They successfully distinguished control (non-meditative state) and meditation states with machine learning algorithms. Karydis et al. [36] identified the post-meditation perceptual states by using a wearable EEG measurement device (Muse headband). Mason et al. [37] examined the effect of yoga on physiological signals. They used PortaPres Digital Plethtsmograph for measuring blood pressure and respiration signals. They also showed the positive effect of yoga by using these signals. A further study validated the positive effect of yoga with physiological signals; researchers monitored breathing and heart rate pulse with a piezoelectric belt and a pulse sensor [21]. They demonstrated the effectiveness of different yogic breathing patterns to help participants relax. There are also several studies showing the effectiveness of mobile mindfulness apps by using physiological signals [20,38,39]. Svetlov et al. [20] monitored the heart rate variability (HRV), electrodermal activity (EDA), Salivary alpha-amylase (sAA) and EEG values. In other studies, EEG and respiration signals were also used for validating the effect of mobile mindfulness apps [38,39]. When the literature is examined, it could be observed that the effect of ancient relaxation methods and mobile mindfulness methods are examined separately in different studies. Ancient methods generally require out of office environments that are not suitable for most of the population, since, in the modern age, people started to spend more time in office-like environments. On the other hand, some smartphone applications such as Pause, HeartMath and Calm do not require extra hardware or equipment and be applicable in office environments. Hence, an ideal solution depends on the context of individuals. A system that monitors stress levels, analyzes the context of individuals and suggests an appropriate relaxation method in the case of high stress will benefit society. Furthermore, mobile methods along with the ancient techniques should be applied in stressful real-life events and their effectiveness should be compared by investigating physiological signals. When the literature is examined, there is not any study comparing the performance of these methods in real-life events (see Table 1). Another important finding is that these methods should be compared with unobtrusive wearable devices so that they could be used for a biofeedback system in daily lives. Individuals may be reluctant to use a system with cables, electrodes and boards in their daily life. Therefore, a comparison of different states with such systems could not be used in daily life. There is clearly a need for a suggestion and comparison of ancient and mobile meditation methods by using algorithms that could run on unobtrusive devices. An ideal system should detect high stress levels, suggest relaxation methods and control whether users are doing these exercises right or not with unobtrusive devices. Our algorithm is suitable to be embedded in such daily life applicable systems that use physiological signals such as skin temperature (ST), HRV, EDA and accelerometer (ACC). In this paper, we present the findings of our pilot study that tested the use of our algorithm during general daily activities, stress reduction activities and a stressful event.

## 4. Methodology

### 4.1. Unobtrusive Stress Detection System with Smart Bands

Our stress detection system developed in [32] allows users to be aware of their stress levels during their daily activities without creating any interruption or restriction. The only requirement to use this system is the need to wear a smart band. Participants in this study wore the Empatica E4 smart band on their non-dominant hand. The smart band provides Blood Volume Pressure, ST, EDA, IBI (Interbeat Interval) and 3D Acceleration. The data are stored in the memory of the device. Then, the artifacts of physiological signals were detected and handled. The features were extracted from the sensory signals and fed to the machine learning algorithm for prediction. In order to use this system, pre-trained machine learning models are required. For training the models, feature vectors and collected class labels were used.

#### 4.1.1. EDA Preprocessing Artifact Detection and Removal Methods

The body sweats when emotional arousal and stress are experienced and, therefore, skin conductance increases [40]. This makes EDA a promising candidate for stress level detection. Intense physical activity and temperature changes contaminate the SC (Skin Conductance) signal. Therefore, affected segments (artifacts) should be filtered out from the original signal. In order to detect the artifacts in the SC signal, we used an EDA toolkit [41] which is 95% accurate on the detection of the artifacts. While developing this tool, technicians labeled the artifacts manually. They trained a machine learning model by using the labels. In addition to the SC signal, 3D acceleration and ST signals were also used for artifact detection. We removed the parts that this tool detected as artifacts from our signals. We further added batch processing and segmentation to this tool by using custom software built-in Python 2.7.

#### 4.1.2. EDA Feature Extraction Methods

After the artifact removal phase, features were extracted from the EDA signal. This signal has two components phasic and tonic; features from both components were extracted (see Table 2). The cvxEDA tool [42] was used for the decomposition of the signal into these components. This tool uses convex optimization to estimate the Autonomic Nervous System (ANS) activity that is based on Bayesian statistics.

##### Tonic Component Features

The tonic component in the EDA signal represents the long-term slow changes. This component is also known as the skin conductance level. It could be regarded as the indicator of general psychophysiological activation [43].

##### Phasic Component Features

The phasic component represents faster (event-related ) differences in the SC signal. The Peaks of phasic SC component as a reaction to a stimulus is also called Skin Conductance Response [43]. After we decompose the phasic component from the EDA signal, peak related features were extracted.

#### 4.1.3. Heart Activity Preprocessing (Artifact Detection and Removal) and Feature Extraction Methods

Heart activity (or, more specifically, HRV) reacts to changes in the autonomic nervous system (ANS) caused by stress [44] and it is, therefore, one of the most commonly used physiological signal for stress detection [40]. However, vigorous movement of subjects and improperly worn devices may contaminate the HRV signal collected from smartwatches and smart bands. In order to address this issue, we developed an artifact handling tool in MATLAB programming language [45] that has batch processing capability. First, the data were divided into 2 min long segments with 50% overlapping. Two-minute segments were selected because it is reported that the time interval for stress stimulation and recovery processes is around a few minutes [46]. The artifact detection percentage rule (also employed in Kubios [47]) was applied after the segmentation phase. In this rule, each data point was compared with the local average around it. When the difference was more than a predetermined threshold percentage, (20% is commonly selected in the literature [48]), the data point was labeled as an artifact. In our system, we deleted the inter-beat intervals detected as the artifacts and interpolated these points with the cubic spline interpolation technique which was used in the Kubios software [47]. The time-domain features of HRV are calculated. In order to calculate the frequency domain features, we interpolated the RR intervals to 4 Hz. Then, we applied the Fast Fourier Transform (FFT). These time and frequency domain features (see Table 3) were selected because these are the most discriminative ones in the literature [30,49,50].

#### 4.1.4. Accelerometer Feature Extraction Methods

Research has shown that movements of the human body and postures can indeed be employed as a means to detect signs of different emotional states. The dynamics of body movement were investigated by Castellano et al. who used multimodal data to identify human affective behaviors. Specific movement metrics, such as the amount of movement, intensity and fluidity, were used to help deduct emotions, and it was found that the amount of movement was a major factor in distinguishing different types of emotions [51]. Melzer et al. investigated whether movements comprised of collections of Laban movement components could be recognized as expressing basic emotions [52]. The results of their study confirm that, even when the subject has no intention of expressing emotions, particular movements can assist in the perception of bodily expressions of emotions. Accelerometer sensors may be used to detect these movements and different types of affect. The accelerometer sensor data are used for two different purposes in our system. Firstly, we extracted features from the accelerometer sensor, for detecting stress levels. We also selected the features to be used as described in Table 4 [53] and, as mentioned above, this sensor was also employed to clean the EDA signal in the EDAExplorer Tool [41].

#### 4.1.5. Skin Temperature

A skin temperature signal is used for the artifact detection phase of the EDA signal in the EDAExplorer Tool [41]. After we divide our data into segments, different modalities were merged into one feature vector. The heart activity signal started with a delay (to calculate heartbeats per minute at the start) and all signals were then synchronized. We included start and end timestamps for each segment, and each modality was merged with a custom Python script.

#### 4.1.6. Machine Learning Classifier Algorithms

The Weka machine learning toolkit [54] is used for identifying stress levels. The Weka toolkit has several preprocessing features before classification. Our data set was not balanced when the number of instances belonging to each class was considered. We solved this issue by removing samples from the majority class. We selected random undersampling because it is the most commonly applied method [55]. In this way, we prevented classifiers from biasing towards the class with more instances. In this study, we employed five different machine learning classification algorithms to recognize different stress levels: MultiLayer Perceptron (MLP), Random Forest (RF) (with 100 trees), K-nearest neighbors (kNN) (*n* = 1–4), Linear discriminant analysis (LDA), Principal component analysis (PCA) and support vector machine (SVM) with a radial basis function. These algorithms were selected because they were the most commonly applied and successful classifiers for detecting stress levels [30,48]. In addition, 10-fold stratified cross-validation was then applied and hyperparameters of the machine learning algorithms were fine-tuned with grid search. The best performing models have been reported.

#### 4.1.7. Dimensionality Reduction

We applied correlation-based feature selection (CBFS) technique which is available in the Weka machine learning package for combined signal [56]. The CBFS method removes the features that are less correlated with the output class. For every model, we selected the ten most important features. This method is applied for MLP, RF, kNN and LDA. In order to create an SVM based model, we applied PCA based dimensionality reduction where the covered variance is selected as 0.95 (the default setting).

#### 4.1.8. Insights from the Feature Selection Process

**Figure 1:**
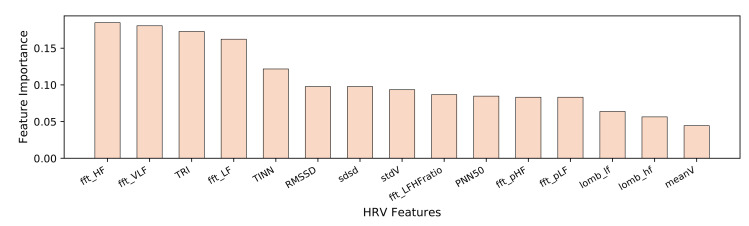
Top-ranking features selected for the HRV signal.

**Figure 2:**
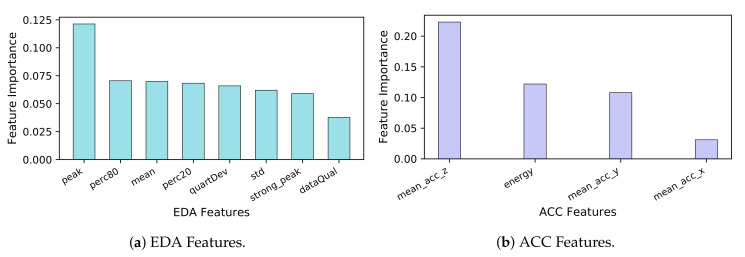
Top-ranking features selected for the EDA and ACC signals.

The CBFS method computes the correlation of features with the ground truth label of the stress level. Insights about the contribution of the features to the stress detection performance can be obtained from Figure 1 and Figure 2. Three of the best features (over 0.15 correlation) are frequency domain features. These features are high, low and very-low frequency components of the HRV signal (see Figure 1). When we examine the EDA features, peaks per 100 s feature are the most important and distinctive feature by far. Since the EDA signal is distorted under the influence of the stimuli, the number of peaks and valleys increases. Lastly, when the acceleration signal is investigated, the most discriminative feature is mean acceleration in the *z*-axis (see Figure 2b). This could be due to the nature of hand and body gestures which are caused by stressed situations.

### 4.2. Relaxation Method Suggestion by Analyzing the Physical Activity-Based Context

Context is a broad term that could contain different types of information such as calendars, activity type, location and activity intensity. Physical activity intensity could be used to infer contextual information. In more restricted environments such as office, classrooms, public transportation and physical activity intensity could be low, whereas, in outdoor environments, physical activity intensity could increase. Therefore, an appropriate relaxation method will change according to the context of individuals.

**Figure 3:**
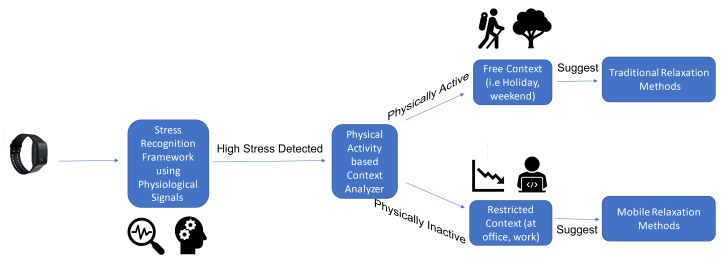
The whole system diagram is depicted. When a high stress level is experienced, by analyzing the physical activity based context, the system suggests the most appropriate reduction method.

For calculating physical activity intensity, we used the EDAExplorer tool [41]. The stillness metric is used for this purpose. It is the percentage of periods in which the person is still or motionless. Total acceleration must be less than a threshold (default is 0.1 [41]) for 95 percent of a minute in order for this minute to count as still [41]. Then, the ratio of still minutes in a session can be calculated. For the ratio of still minutes in a session, we labeled sessions below 20% as still, above 20% as active and suggested relaxation method accordingly (see Figure 3).

### 4.3. Description of the Data Collection Procedure

The proposed stress level monitoring mechanism, for real-life settings, was evaluated during an eight day Marie Skłodowska-Curie Innovative Training Network (ITN) training event in Istanbul, Turkey, for the AffecTech project. AffecTech is a program funded by Horizon 2020 (H2020) framework established by the European Commission. The AffecTech project is an international collaborative research network involving 15 PhD students (early stage researchers (ESR)) with the aim of developing low-cost effective wearable technologies for individuals who experience affective disorders (for example, depression, anxiety and bipolar disorder).

**Figure 4:**
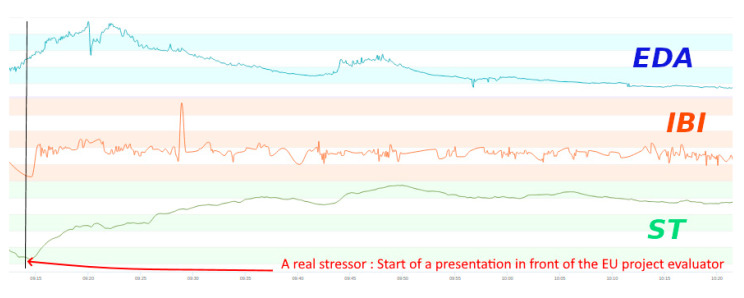
Sample data belong to a presentation session. The increase in EDA, ST and IBI could be observed when the subject started the presentation.

The eight-day training event included workshops, lectures and training with clearly defined tasks and activities to ensure that the ESR had developed the required skills, knowledge and values outline prior to the training event. At the end of the eight-day training, ESRs were required to deliver a presentation about their PhD work to two evaluators from the European Union where they received feedback about their progress (see Figure 4 for raw physiological signals at the start of the presentation). For studying the effects of emotion regulation on stress, yoga, guided mindfulness and mobile-based mindfulness, sessions were held by a certified instructor.

During the training, physiological and questionnaire data were collected from the 16 ESR participants (9 men, mean age 28); 15 ESRs and one of the AffecTech project academics, all of whom gave informed consent to participate in the study. Participants were from different countries with diverse nationalities (two from Iran, two from Spain, two from Italy, one from Argentina, one from Pakistan, one from China, one from Switzerland, one from Belarus, one from France, one from England, one from Barbados, one from Turkey and one from Bulgaria). Due to the fault of one of the Empatica E4 devices, it was not possible to include data from one participant. The remaining 15 participants completed all stages of the study successfully.

**Figure 5:**
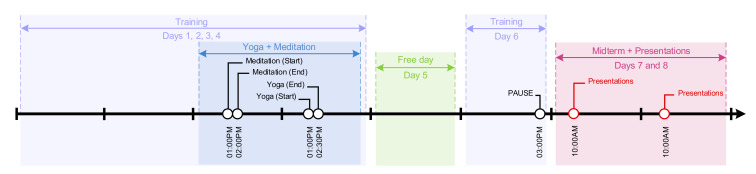
Time-line depicting eight days of the training event. Presentations, relaxations and lectures are highlighted.

During the eight days of training and presentations, psychophysiological data were collected from 16 participants during the training event from Empatica E4 smart band while they are awake. For studying the effects of emotion regulation on stress, yoga, guided mindfulness and mobile-based mindfulness sessions were held by a certified instructor. The timeline of the event is shown in Figure 5.

#### 4.3.1. Physiological Stress Data

The psychophysiological signal data were collected using the Empatica E4 smart band whilst participants were awake throughout the eight days of the AffecTech training. Physiological data included IBI, EDA, ACC (Accelerometer) and ST and stored in different csv files. In addition, 27.39% of the data are obtained from free times (free day and after training until subjects slept 5:00 p.m.–10:00 p.m.), 43.83% of the data comes from lectures in the training, 11.41% is the presentation session and relax sessions consist of 17.35% of the data. As mentioned previously, we randomly undersampled (most commonly applied method [55] ) the data to overcome the class imbalance problem. The participants’ blood pressure (BP) was also recorded using CE(0123) Harvard Medical Devices Ltd. automated sphygmomanometer prior to and after each stress reduction event (yoga and mindfulness), in order to demonstrate whether the participants stress levels were modified. On each occasion that the participants’ BP was recorded, the mean of three recordings was used as the final BP. A reduction in the participants’ blood pressure and/or pulse rate may be seen, which demonstrates a reduction in stress level.

#### 4.3.2. Ethics

The procedure used in this study was approved by the Institutional Review Board for Research with Human Subjects of Boğaziçi University with the approval number 2018/16. Prior to data acquisition, each participant received a consent form describing the experimental procedure and its benefits and implications to both the society and the subject. The procedure was also explained verbally to the subject. All of the data are stored anonymously.

#### 4.3.3. Questionnaire Self-Report Stress Data

A session-based self-report questionnaire comprised of six questions based on the Nasa Task Load Index (NASA-TLX) [57]. The frustration scale was specifically used to measure perceived stress levels [32]. We asked the following question to the participants for each session:

How irritated, stressed and annoyed versus content, relaxed and complacent did you feel during the task?

Questionnaires were completed daily (at the end of the day) and, after each presentation, lecture and stress reduction event (such as yoga and mindfulness).

#### 4.3.4. Stress Management Scheme Using Yoga and Mindfulness

During the eight day training, it is assumed that the participants’ stress levels are likely to have increased day by day because they were required to give a presentation (perceived as a stressful event) reporting their PhD progress to the EU project evaluators at the end of the training.

**Figure 6:**
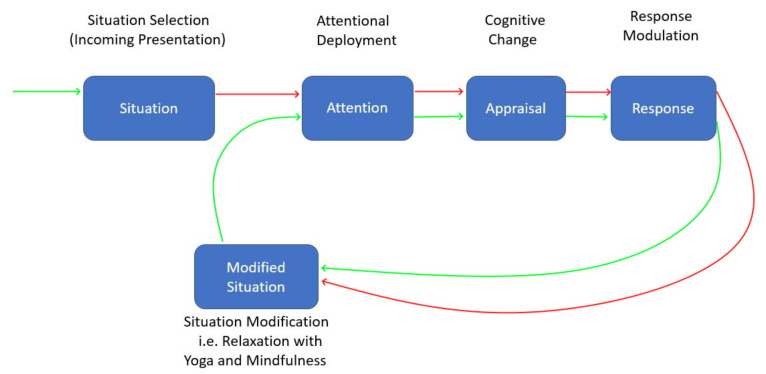
Application of James Gross’s Emotion Regulation model [4] in the context of stress management.

Underpinned by James Gross’s Emotion Regulation model (see Figure 6) [4], we modified the situation to help the participants to reduce their thoughts of the end of the training presentation. To help participants manage their stress levels, we applied Yoga and mindfulness sessions on two separate days (day three and day four, respectively). These sessions lasted approximately 1 h and, throughout the sessions, participants wore an Empatica E4 smartband. In addition to the physiological signals coming from the Smartbands, participants’ blood pressure values were also recorded before and after the yoga and mindfulness sessions.

## 5. Experimental Results and Discussion

### 5.1. Statistical Data Analysis

#### 5.1.1. Validation of Different Perceived Stress Levels by using the Self-Reports

**Figure 7:**
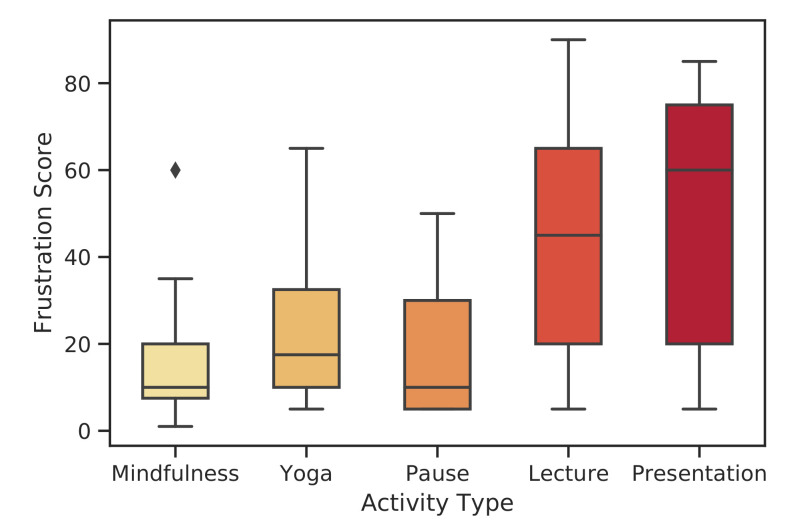
Visual representation of the frustration scores collected in different types of sessions.

In order to validate that the participants experienced different perceived stress levels in different contexts (lecture, relaxation, presentation), we used the Frustration item (see Section 4.5) from the NASA-TLX [57]. The distribution of answers is demonstrated in Figure 7. Our aim is to show that the perceived stress levels (obtained from self-report answers) differ in relaxation sessions considerably when compared to the presentation session (high stress). To this end, we applied the *t*-test (in R programming language) to the perceived stress self-report answers of yoga versus presentation, mindfulness versus presentation and pause (mobile mindfulness) versus presentation session pairs. The paired *t*-test is used to evaluate the separability of each session. The degree of freedom is 15. We applied the variance test to each session tuple; we could not identify equal variance in any of the session tuples. Thus, we selected the variance as unequal. We used 99.5% confidence intervals. The *t*-test results’ (*p*-values and test statistics) are provided in Table 5. For all tuples, the null hypothesis stating that the perceived stress of the relaxation method is not less than the presentation session is rejected. The perceived stress levels of participants for all meditation sessions are observed to be significantly lower than the presentation session (high stress).

#### 5.1.2. Before and After Physiological Measurements for Evaluating Performance of Yoga and Mindfulness with Blood Pressure

In this section, we compared the effect of stress management tools such as yoga and mindfulness on blood pressure. It is expected that blood pressure sensors will be part of unobtrusive wrist-worn wearable sensors soon. We plan to integrate a blood pressure (BP) module to our system when they are available. Therefore, by using the measurements of a medical-grade blood pressure monitor, we provided insights about how stress reaction affects BP. We further applied and tested the prominent emotion regulation model of James Gross by analyzing these measurements in the context of stress management. We measured the diastolic and systolic BP and pulse using a medical-grade blood pressure monitor before and after the yoga and mindfulness sessions. In order to ensure that the participants were relaxed and that an accurate BP was recorded, BP was measured three times with the mean as the recorded result. A one-sample *t*-test was applied to the difference between mean values. The results are shown in Table 6.

Mindfulness decreased the systolic BP, –1.13% (ns), increased diastolic BP, +1.75% (*p* < 0.05) and decreased the pulse –5.75% (*p* < 0.05). Medicine knows that systolic blood pressure (the top number or highest blood pressure when the heart is squeezing and pushing the blood around the body) is more important than diastolic blood pressure (the bottom number or lowest blood pressure between heartbeats) because it gives the best idea of the risk of having a stroke or heart attack. In this view, the significant reduction of systolic BP after mindfulness is an important result.

Moreover, the difference between systolic and diastolic BP is called pulse pressure. For example, 120 systolic minus 60 diastolic equals a pulse pressure of 60. It is also known that a pulse pressure greater than 60 can be a predictor of heart attacks or other cardiovascular diseases, while a low pulse pressure (less than 40) may indicate poor heart function. In our study, pulse pressure was lower after mindfulness (we had both a significant reduction in systolic BP and an increase in diastolic BP), but its value was higher than 40 (42.69 mean difference before the mindfulness and 40.48 mean difference after the mindfulness), suggesting that this result can also be considered clinically positive.

During yoga, there was a decrease in systolic BP by −5.81% (*p* < 0.05), diastolic BP by −1.93% (ns) and increase in pulse +8.06% (*p* < 0.05). Yoga appears to be more effective than mindfulness at decreasing systolic and diastolic blood pressure, although mindfulness seems to be more effective than yoga for decreasing the pulse due to the activity involved in yoga.

### 5.2. Physiological Stress Level Detection with Wearables by Using Context Labels as the Class Label

We tested our system by using the known context labels of sessions as the class label. We used Lecture (mild stress), Yoga and Mindfulness (relax) and Presentation in front of the board of juries (high stress) as class labels by examining perceived stress self-report answers in Figure 6. We investigated the success of relaxation methods, different modalities and finding the presenter.

#### 5.2.1. Effect of Different Physiological Signals on Stress Detection

We evaluated the effect of using the interbeat-interval, the skin conductance and the accelerometer signals separately and in a combined manner on two and three class classification performance. These classes are mild stress, high stress and relax states from mindfulness and yoga sessions. The results are shown in Table 7, Table 8 and Table 9. For the three-class classification problem, we achieved a maximum accuracy of 72% by using MLP on only HRV features and 86.61% with only accelerometer features using the Random Forest classifier and 85.36% accuracy combination of all features with LDA classifier (see Table 7). The difficulty in this classification task is a similar physiological reaction to relax and mild stress situations. However, since the main focus of our study is to discriminate high stress from other classes to offer relaxation techniques in this state, it did not affect our system performance. We also investigated high-mild stress and high stress-relax 2-class classification performance. For the discrimination of high and mild stress, HRV outperformed other signals with 98% accuracy using MLP (see Table 8). In the high stress-relax 2-class problem, only HRV features with RF achieved a maximum accuracy of 86%, whereas ACC features with MLP achieved a maximum of 94% accuracy. In this problem, the combination of all signals with RF achieved 92% accuracy which is the best among all classifiers (see Table 9). For all models, EDA did not perform well. This might be caused by the loose contact with EDA electrodes in the strap due to loosely worn smartbands.

#### 5.2.2. Effectiveness of Yoga, Mindfulness and Mobile Mindfulness (Pause)

We applied three different relaxation methods to manage stress levels of individuals. In order to measure the effectiveness of each method, we examined how easily these physiological signals in the relaxation sessions can be separated from high stress presentations. If it can be separated from high stress levels with higher classification performance, it could be inferred that they are more successful at reducing stress. As seen in Table 10 and Table 11, mobile mindfulness has lower success in reducing stress levels. Yoga has the highest classification performance with both HR and EDA signals.

## 6. Conclusions

In this study, by using our automatic stress detection system with the use of Empatica-E4 smart-bands, we detected stress levels and suggested appropriate relaxation methods (i.e., traditional or mobile) when high stress levels are experienced. Our stress detection framework is unobtrusive, comfortable and suitable for use in daily life and our relaxation method suggestion system makes its decisions based on the physical activity-related context of a user. To test our system, we collected eight days of data from 16 individuals participating in an EU research project training event. Individuals were exposed to varied stressful and relaxation events (1) training and lectures (mild stress), (2) yoga, mindfulness and mobile mindfulness (PAUSE) (relax) and (3) were required to give a moderated presentation (high stress). The participants were from different countries with diverse cultures.

In addition, 1440 h of mobile data (12 h in a day) were collected during this eight-day event from each participant measuring their stress levels. Data were collected during the training sessions, relaxation events and the moderated presentation and during their free time for 12 h in a day, demonstrating that our study monitored daily life stress. EDA and HR signals were collected to detect physiological stress and a combination of different modalities increased stress detection, performance and provided the most discriminative features. We first applied James Gross ER model in the context of stress management and measured the blood pressure during the ER cycle. When the known context was used as the label for stress level detection system, we achieved 98% accuracy for 2-class and 85% accuracy for 3-class. Most of the studies in the literature only detect stress levels of individuals. The participants’ stress levels were managed with yoga, mindfulness and a mobile mindfulness application while monitoring their stress levels. We investigated the success of each stress management technique by the separability of physiological signals from high-stress sessions. We demonstrated that yoga and traditional mindfulness performed slightly better than the mobile mindfulness application. Furthermore, this study is not without limitations. In order to generalize the conclusions, more experiments based on larger sample groups should be conducted. As future work, we plan to develop personalized perceived stress models by using self-reports and test our system in the wild. Furthermore, attitudes in the psychological field constitute a topic of utmost relevance, which always play an instrumental role in the determination of human behavior [58]. We plan to design a new experiment which accounts for the attitudes of participants towards relaxation methods and their effects on the performance of stress recognition systems.

## Figures and Tables

**Table 1:** Comparison of our work with the studies applying different types of meditation techniques for stress management in the literature.

Article	YOGA	Mindfulness	Mobile Relaxation	Device	Signal	Daily Suitable
Ahani et al. [35]	X	🗸	X	Biosemi	EEG and Respiration	No
Mason et al. [37]	🗸	X	X	Digital Plethysmograph (PortaPres)	Virtual Blood Pressure Respiration	No
Svetlov et al. [20]	X	X	🗸	Several	HRV, EDA, sAA and EEG	No
Puranik et al. [21]	🗸	X	X	MPU 6050 + piezoelectric belt + pulse sensor + smartphone	Heart Rate + Respiration	No
Karydis et al. [36]	X	🗸	X	Muse Headband	EEG	No
Cheng et al. [38]	X	X	🗸	Emotiv wireless headset	EEG	No
Ingle et al. [39]	X	X	🗸	8-channel Enobio EEG + piezoelectric belt	EEG + Respiratory	No
Our work	🗸	🗸	🗸	Empatica E4 wristband	PPG (Photoplethysmography), EDA, ACC, ST	Yes

**Table 2:** EDA features and their definitions.

Feature	Description
Quartdev Tonic	Quartile deviation (75 percentile–25 percentile) of the phasic component
Strong Peaks Phasic	The number of strong peak per 100 s
Peaks Phasic	The number of peaks per 100 s
Perc20	20th percentile of the phasic component
Perc80 Tonic	80th percentile of the phasic component
Mean Tonic	Mean of the phasic component
SD Tonic	Standard deviation of phasic component

**Table 3:** HRV features and their definitions [32].

Feature	Description
Mean RR	Mean value of the inter-beat (RR) intervals
STD RR	Standard deviation of the inter-beat interval
pNN50	Percentage of the number of successive RR intervals varying more than 50 ms from the previous interval
RMSSD	Root mean square of successive difference of the RR intervals
SDSD	Related standard deviation of successive RR interval differences
HRV triangular index	Total number of RR intervals divided by the height of the histogram of all RR intervals measured on a scale with bins of 1/128 s
TINN	Triangular interpolation of RR interval histogram
LF	Power in low-frequency band (0.04–0.15 Hz)
HF	Power in high-frequency band (0.15–0.4 Hz)
pLF	Prevalent low-frequency oscillation of heart rate
pHF	Prevalent high-frequency oscillation of heart rate
VLF	Power in very low-frequency band (0.00–0.04 Hz)
LF/HF	Ratio of LF-to-HF

**Table 4:** ACC features and their definitions.

Feature	Description
Mean X	Mean acceleration over *x* axis
Mean Y	Mean acceleration over *y* axis
Mean Z	Mean acceleration over *z* axis
MeanAccMag	Mean acceleration over acceleration magnitude
Energy	FFT energy over mean acceleration magnitude

**Table 5:** *T*-test results for session tuple comparison of perceived stress levels using self-reports.

Session Tuple	*t*-Test Statistic	*p*-Value
Yoga—Presentation	−4.0027	*p* < 0.005
Guided Mindfulness—Presentation	−5.4905	*p* < 0.005
Mobile Mindfulness—Presentation	−4.2677	*p* < 0.005

**Table 6:** The difference between the mean diastolic blood pressure, the mean systolic blood pressure and the mean pulse, before and after sessions of guided mindfulness and guided yoga. (* *p* < 0.05).

Activity	Systolic	Diastolic	Pulse
Guided Mindfulness	−1.31%	1.75% *	−5.75% *
Guided Yoga	−5.81% *	−1.93%	8.06% *

**Table 7:** Effect of different modalities and their combination on the system performance. Note that the number of classes is fixed at 3 (high stress, mild stress and relax).

Algorithm	Accuracy, %			
	HRV	EDA	ACC	Combined
MLP	72.14	36.61	74.29	82.68
RF	67.86	36.96	86.61	85.18
kNN	65.00	29.82	70.89	78.39
LDA	69.82	31.96	73.39	85.36
SVM	47.14	30.54	58.57	46.96

**Table 8:** Effect of different modalities and their combination on the system performance. Note that the number of classes is fixed at 2 (high stress and mild stress).

Algorithm	Accuracy, %			
	HRV	EDA	ACC	Combined
MLP	98.00	60.00	64.00	98.00
RF	98.00	42.00	72.00	98.00
kNN	94.00	44.00	58.00	94.00
LDA	94.00	40.00	54.00	94.00
SVM	66.00	54.00	54.00	66.00

**Table 9:** Effect of different modalities and their combination on the system performance. Note that the number of classes is fixed at 2 (high stress and relax).

Algorithm	Accuracy, %			
	HRV	EDA	ACC	Combined
MLP	82.00	66.00	96.00	90.00
RF	86.00	60.00	94.00	92.00
kNN	82.00	66.00	88.00	90.00
LDA	78.00	64.00	92.00	88.00
SVM	78.00	62.00	52.00	74.00

**Table 10:** The classification accuracy of the relaxation sessions using stress management methods and stressful sessions using EDA.

Algorithm	Accuracy, %		
	Guided Mindfulness	Yoga	Mobile Mindfulness
MLP	65.71	78.57	75.00
RF	67.14	87.14	67.64
kNN	64.29	82.86	77.94
LDA	65.71	80.00	51.47
SVM	70.00	72.86	58.82

**Table 11:** The classification accuracy of the relaxation sessions using stress management methods and stressful sessions using HRV.

Algorithm	Accuracy, %		
	Guided Mindfulness	Yoga	Mobile Mindfulness
MLP	90.00	97.50	93.94
RF	97.50	95.00	87.89
kNN	90.00	90.00	93.93
LDA	87.50	87.50	75.75
SVM	85.00	80.00	81.82

## References

[1] Bali A., Jaggi A.S. (2015). Clinical experimental stress studies: Methods and assessment. Rev. Neurosci..

[2] Ingram R.E., Luxton D.D. (2005). Vulnerability-stress models. Development of Psychopathology: A Vulnerability-Stress Perspective.

[3] Harkness K.L., Hayden E.P. (2018). The Oxford Handbook of Stress and Mental Health.

[4] Gross J.J. (1998). The emerging field of emotion regulation: An integrative review. Rev. Gen. Psychol..

[5] Koole S.L., Aldao A. (2016). The self-regulation of emotion: Theoretical and empirical advances. Handbook of Self-Regulation: Research, Theory and Applications.

[6] Dimsdale J.E. (2008). Psychological Stress and Cardiovascular Disease. J. Am. Coll. Cardiol..

[7] Fink G. (2010). Stress: Concepts, Definition and History. 2017. Fink, G. Stress: Definition and history. Stress Science: Neuroendocrinology.

[8] (2019). Exercise: A Guide to Tai Chi. https://www.nhs.uk/live-well/exercise/guide-to-tai-chi.

[9] Chong C.S., Tsunaka M., Chan E.P. (2011). Effects of yoga on stress management in healthy adults: A systematic review. Altern. Ther. Health Med..

[10] Asmundson G.J., Fetzner M.G., DeBoer L.B., Powers M.B., Otto M.W., Smits J.A. (2013). Let’s get physical: A contemporary review of the anxiolytic effects of exercise for anxiety and its disorders. Depress. Anxiety.

[11] Song Y., Lindquist R. (2015). Effects of mindfulness-based stress reduction on depression, anxiety, stress and mindfulness in Korean nursing students. Nurse Educ. Today.

[12] Arch J.J., Ayers C.R., Baker A., Almklov E., Dean D.J., Craske M.G. (2013). Randomized clinical trial of adapted mindfulness-based stress reduction versus group cognitive behavioral therapy for heterogeneous anxiety disorders. Behav. Res. Ther..

[13] Cheng P., Lucero A., Buur J. (2016). PAUSE: Exploring Mindful Touch Interaction on Smartphones. Proceedings of the 20th International Academic Mindtrek Conference (AcademicMindtrek’16).

[14] Harkness K., Hayden E., Olino T., Mennies R., Wojcieszak Z. (2020). Personality-Stress Vulnerability Models. The Oxford Handbook of Stress and Mental Health.

[15] McEwen B.S. (2005). Stressed or stressed out: What is the difference?. J. Psychiatry Neurosci..

[16] Aldao A., Nolen-Hoeksema S. (2012). The influence of context on the implementation of adaptive emotion regulation strategies. Behav. Res. Ther..

[17] Troy A.S., Mauss I.B. (2011). Resilience in the face of stress: Emotion regulation as a protective factor. Resil. Ment. Heal. Chall. Across Lifesp..

[18] Mennin D.S., Fresco D.M., Ritter M., Heimberg R.G. (2015). An open trial of emotion regulation therapy for generalized anxiety disorder and cooccurring depression. Depress. Anxiety.

[19] Radkovsky A., McArdle J.J., Bockting C.L.H., Berking M. (2014). Successful emotion regulation skills application predicts subsequent reduction of symptom severity during treatment of major depressive disorder. J. Consult. Clin. Psychol..

[20] Svetlov A.S., Nelson M.M., Antonenko P.D., McNamara J.P., Bussing R. (2019). Commercial mindfulness aid does not aid short-term stress reduction compared to unassisted relaxation. Heliyon.

[21] Puranik K.A., Kanthi M. Wearable Device for Yogic Breathing. Proceedings of the 2019 Amity International Conference on Artificial Intelligence (AICAI).

[22] NICE (2009). Depression in Adults: Recognition and Management. Clinical Guideline [CG90].

[23] Huston P., McFarlane B. (2016). Health benefits of tai chi. Can. Fam. Physician.

[24] PauseAble—Mindfulness in Motion. https://www.pauseable.com/.

[25] Castaldo R., Montesinos L., Melillo P., Massaro S., Pecchia L. (2018). To What Extent Can We Shorten HRV Analysis in Wearable Sensing? A Case Study on Mental Stress Detection. EMBEC & NBC 2017.

[26] Fernández J.R.M., Anishchenko L. (2018). Mental stress detection using bioradar respiratory signals. Biomed. Signal Process. Control.

[27] Giannakakis G., Pediaditis M., Manousos D., Kazantzaki E., Chiarugi F., Simos P.G., Marias K., Tsiknakis M. (2017). Stress and anxiety detection using facial cues from videos. Biomed. Signal Process. Control.

[28] Castaldo R., Xu W., Melillo P., Pecchia L., Santamaria L., James C. Detection of mental stress due to oral academic examination via ultra-short-term HRV analysis. Proceedings of the 2016 38th Annual International Conference of the IEEE Engineering in Medicine and Biology Society (EMBC).

[29] Vildjiounaite E., Kallio J., Kyllönen V., Nieminen M., Mäntyjärvi J., Gimel’farb G. (2018). Unobtrusive stress detection on the basis of smartphone usage data. Pers. Ubiquitous Comput..

[30] Gjoreski M., Luštrek M., Gams M., Gjoreski H. (2017). Monitoring stress with a wrist device using context. J. Biomed. Inform..

[31] Gjoreski M., Gjoreski H., Luštrek M., Gams M. (2016). Continuous Stress Detection Using a Wrist Device: In Laboratory and Real Life. Proceedings of the 2016 ACM International Joint Conference on Pervasive and Ubiquitous Computing: Adjunct (UbiComp’16).

[32] Can Y.S., Chalabianloo N., Ekiz D., Ersoy C. (2019). Continuous Stress Detection Using Wearable Sensors in Real Life: Algorithmic Programming Contest Case Study. Sensors.

[33] Ciman M., Wac K. (2016). Individuals’ stress assessment using human-smartphone interaction analysis. IEEE Trans. Affect. Comput..

[34] Sysoev M., Kos A., PogaăźNik M. (2015). Noninvasive Stress Recognition Considering the Current Activity. Pers. Ubiquitous Comput..

[35] Ahani A., Wahbeh H., Miller M., Nezamfar H., Erdogmus D., Oken B. Change in physiological signals during mindfulness meditation. Proceedings of the 2013 6th International IEEE/EMBS Conference on Neural Engineering (NER).

[36] Karydis T., Langer S., Foster S.L., Mershin A. (2018). Identification of Post-meditation Perceptual States Using Wearable EEG and Self-Calibrating Protocols. Proceedings of the 11th PErvasive Technologies Related to Assistive Environments Conference (PETRA’18).

[37] Mason H., Vandoni M., Debarbieri G., Codrons E., Ugargol V., Bernardi L. (2013). Cardiovascular and respiratory effect of yogic slow breathing in the yoga beginner: What is the best approach?. Evid. Based Complement. Altern. Med..

[38] Pause EEG Validation Article. https://www.ustwo.com/blog/the-story-of-pause.

[39] Ingle R., Awale R. (2018). Impact Analysis of Meditation on Physiological Signals. JOIV Int. J. Inform. Vis..

[40] Can Y.S., Arnrich B., Ersoy C. (2019). Stress Detection in Daily Life Scenarios Using Smart Phones and Wearable Sensors: A Survey. J. Biomed. Inform..

[41] Taylor S., Jaques N., Chen W., Fedor S., Sano A., Picard R. Automatic identification of artifacts in electrodermal activity data. Proceedings of the 2015 37th Annual International Conference of the IEEE Engineering in Medicine and Biology Society (EMBC).

[42] Greco A., Valenza G., Lanata A., Scilingo E.P., Citi L. (2016). cvxEDA: A Convex Optimization Approach to Electrodermal Activity Processing. IEEE Trans. Biomed. Eng..

[43] Kappeler-Setz C. (2012). Multimodal Emotion and Stress Recognition.

[44] Kim H.G., Cheon E.J., Bai D.S., Lee Y.H., Koo B.H. (2018). Stress and heart rate variability: A meta-analysis and review of the literature. Psychiatry Investig..

[45] MATLAB (2018). 9.7.0.1190202 (R2019b).

[46] Stress Response. https://www.anxietycentre.com/anxiety/stress-response.shtml.

[47] Tarvainen M.P., Niskanen J.P., Lipponen J.A., Ranta-aho P.O., Karjalainen P.A., Vander Sloten J., Verdonck P., Nyssen M., Haueisen J. (2009). Kubios HRV—A Software for Advanced Heart Rate Variability Analysis. Proceedings of the 4th European Conference of the International Federation for Medical and Biological Engineering.

[48] Cinaz B., Arnrich B., Marca R., Tröster G. (2013). Monitoring of Mental Workload Levels During an Everyday Life Office-work Scenario. Pers. Ubiquitous Comput..

[49] Alberdi A., Aztiria A., Basarab A. (2016). Towards an automatic early stress recognition system for office environments based on multimodal measurements: A review. J. Biomed. Inform..

[50] Greene S., Thapliyal H., Caban-Holt A. (2016). A Survey of Affective Computing for Stress Detection: Evaluating technologies in stress detection for better health. IEEE Consum. Electron. Mag..

[51] Castellano G., Villalba S.D., Camurri A. Recognising human emotions from body movement and gesture dynamics. Proceedings of the International Conference on Affective Computing and Intelligent Interaction.

[52] Melzer A., Shafir T., Tsachor R.P. (2019). How Do We Recognize Emotion From Movement? Specific Motor Components Contribute to the Recognition of Each Emotion. Front. Psychol..

[53] Tang T.B., Yeo L.W., Lau D.J.H. Activity awareness can improve continuous stress detection in galvanic skin response. Proceedings of the IEEE SENSORS 2014.

[54] Eibe F., Hall M., Witten I. (2016). The WEKA Workbench. Online Appendix for “Data Mining: Practical Machine Learning Tools and Techniques”.

[55] Zhang W., Ramezani R., Naeim A. (2019). WOTBoost: Weighted Oversampling Technique in Boosting for imbalanced learning. arXiv.

[56] Holmes G., Donkin A., Witten I.H. WEKA: A machine learning workbench. Proceedings of the ANZIIS’94— Australian New Zealnd Intelligent Information Systems Conference.

[57] Hart S.G. (1986). NASA Task Load Index (TLX).

[58] Glasman L.R., Albarracin D. (2006). Forming attitudes that predict future behavior: A meta-analysis of the attitude-behavior relation. Psychol. Bull..

